# Automated Analysis of Ultrasound for the Diagnosis of Pneumothorax: A Systematic Review

**DOI:** 10.7759/cureus.72896

**Published:** 2024-11-02

**Authors:** Jonathan Kossoff, Sean Duncan, Jesal Acharya, Daniel Davis

**Affiliations:** 1 Acute Medicine, University College Hospital, London, GBR; 2 Oncology, Digital Health Validation Lab, University of Glasgow, Glasgow, GBR; 3 Geriatrics, Institute of Health Informatics, University College London, London, GBR

**Keywords:** artificial intelligence, automated diagnosis, computer-aided diagnosis, lung sliding, lung ultrasound, pneumothorax, point-of-care ultrasonography, ultrasound

## Abstract

Automated analysis of lung ultrasound for pneumothorax detection is an emerging technology with potential applications across various clinical settings. This systematic review aims to evaluate the current evidence for the efficacy of automated analysis techniques in diagnosing pneumothorax using lung ultrasound images and videos.

A literature search was conducted in the MEDLINE (Medical Literature Analysis and Retrieval System Online), Embase (Excerpta Medica Database), Web of Science, and Scopus databases up to July 5, 2024. Two reviewers screened articles, extracted data, and assessed the risk of bias using a modified QUADAS-2 (the revised version of the Quality Assessment of Diagnostic Accuracy Studies) tool.

Ten studies met the inclusion criteria, with nine published after 2020. Eight studies were retrospective, and nine used physician analysis of ultrasound as the reference standard. Convolutional neural networks were the predominant classification method. The majority of studies analysed the presence or absence of lung sliding. Significant heterogeneity in study designs, outcome metrics, and methodology limited direct comparisons of efficacy.

Automated analysis of lung ultrasound for pneumothorax detection shows promise; however, the field is still in its early stages. Challenges identified include the limitations of lung sliding as a diagnostic feature, variability in ultrasound hardware and protocols, and accounting for low-quality scans. Future research should focus on larger, more diverse datasets, standardized evaluation metrics, and robust external validation.

## Introduction and background

Pneumothorax is a critical diagnosis in a broad range of clinical settings. The diagnostic gold standard has evolved from examination findings to X-rays and the current standard of computed tomography (CT). While all three of these modalities still have a significant role to play, ultrasound is growing in diagnostic significance.

Ultrasound offers the advantages of portability and speed. It is radiation-free. It has become well-established in emergency [1] and intensive care settings [2]. A haemodynamically significant pneumothorax can be excluded by the identification of lung sliding at a single point on the appropriate side. Evidence has shown that ultrasound has superior diagnostic performance for pneumothorax in comparison to chest X-rays [1,2]. Ultrasound machines are increasingly small, with current models capable of running from a smartphone.

A limitation is the difficulty of training. Ultrasound is a user-dependent modality, best interpreted in real-time [3]. Identification or exclusion of pneumothorax requires the analysis of several features. The most significant sonographic feature relevant to the diagnosis of pneumothorax is lung sliding. This represents the sliding of the parietal and visceral pleura against each other. It can be analysed in either B-mode videos or M-mode images. Its presence excludes a pneumothorax at the scanned point [4]. Two other ultrasonographic features that can contribute to the diagnosis of pneumothorax are the lung point and the lung pulse. The lung point represents the point at which pneumothorax and normal lung meet at the pleura and is considered highly specific for pneumothorax. The lung pulse is the rhythmic movement of the pleura with the cardiac cycle and its presence excludes pneumothorax.

Automated techniques for ultrasound analysis, in particular artificial intelligence (AI), have seen an explosion of research interest in the last few years. A broad range of applications have been investigated including echocardiography [5], breast ultrasound [6], musculoskeletal ultrasound [7], and renal ultrasound [8]. Within lung ultrasound, automatic B line recognition [9], pneumonia diagnosis [10], and identification of effusions [11] have all been studied. Developments have been made in supplementary tasks necessary for automated diagnostics in this area, such as anatomical segmentation [12] and identification of the pleural line [13]. The testing of lung sliding classification software in porcine models has shown promising sensitivities and specificities [14,15].

An effective automated diagnostic technique for pneumothorax would have a number of potential uses. For experienced sonographers, it could provide decision support and reduce cognitive load. For learners, it could give confidence in their findings and facilitate their training. In pre-hospital scenarios, it could guide triage and management decisions where access to trained staff is not feasible. Within the hospital, an area of high clinical utility could be reducing the requirement for X-rays following procedures which are at risk of causing pneumothorax.

This systematic review will examine the quality of evidence for the efficacy of automated analysis of lung ultrasound for the diagnosis of pneumothorax.

## Review

Methods

This systematic review was registered on PROSPERO (International Prospective Register of Systematic Reviews) under the code CRD42024549377.

Search Strategy

A literature search was performed in the databases MEDLINE (Medical Literature Analysis and Retrieval System Online), Embase (Excerpta Medica Database), Web of Science, and Scopus. A search strategy was developed in conjunction with a professional librarian. Final searches were conducted on July 5, 2024. A summary of the search strategy is included in the Appendices.

Screening

The inclusion criterion was studies that used automated techniques to analyse ultrasound images or videos to diagnose or exclude pneumothorax.

Studies were excluded if they were animal studies, retracted studies, contained exclusively paediatric research, or were conference proceedings.

After duplicates were removed, both reviewers independently screened the titles and abstracts of studies. The screening was performed using Rayyan.ai software (Rayyan Systems Inc., Cambridge, Massachusetts, United States). After initial screening, the full text of the remaining studies was obtained. Disagreements were resolved by discussion.

Data Extraction

Key characteristics, in terms of study design, testing dataset, and software methodology, were extracted by one author and then checked by another.

Some studies presented the results of multiple models or experiments. In these cases, the statistics and methodology extracted were for the version highlighted in the abstract or the best version as identified by the authors. Statistical outcomes that were extracted were sensitivity, specificity, accuracy, and area under the curve. Due to the heterogenicity of statistical data, no meta-analysis was performed. Narrative synthesis was used to analyse elements of methodology and outcomes.

Quality of bias assessment was performed using a modified version of the QUADAS-2 (the revised version of the Quality Assessment of Diagnostic Accuracy Studies) tool. This was completed independently by two authors. Disagreements were resolved by discussion.

Results

**Figure 1:**
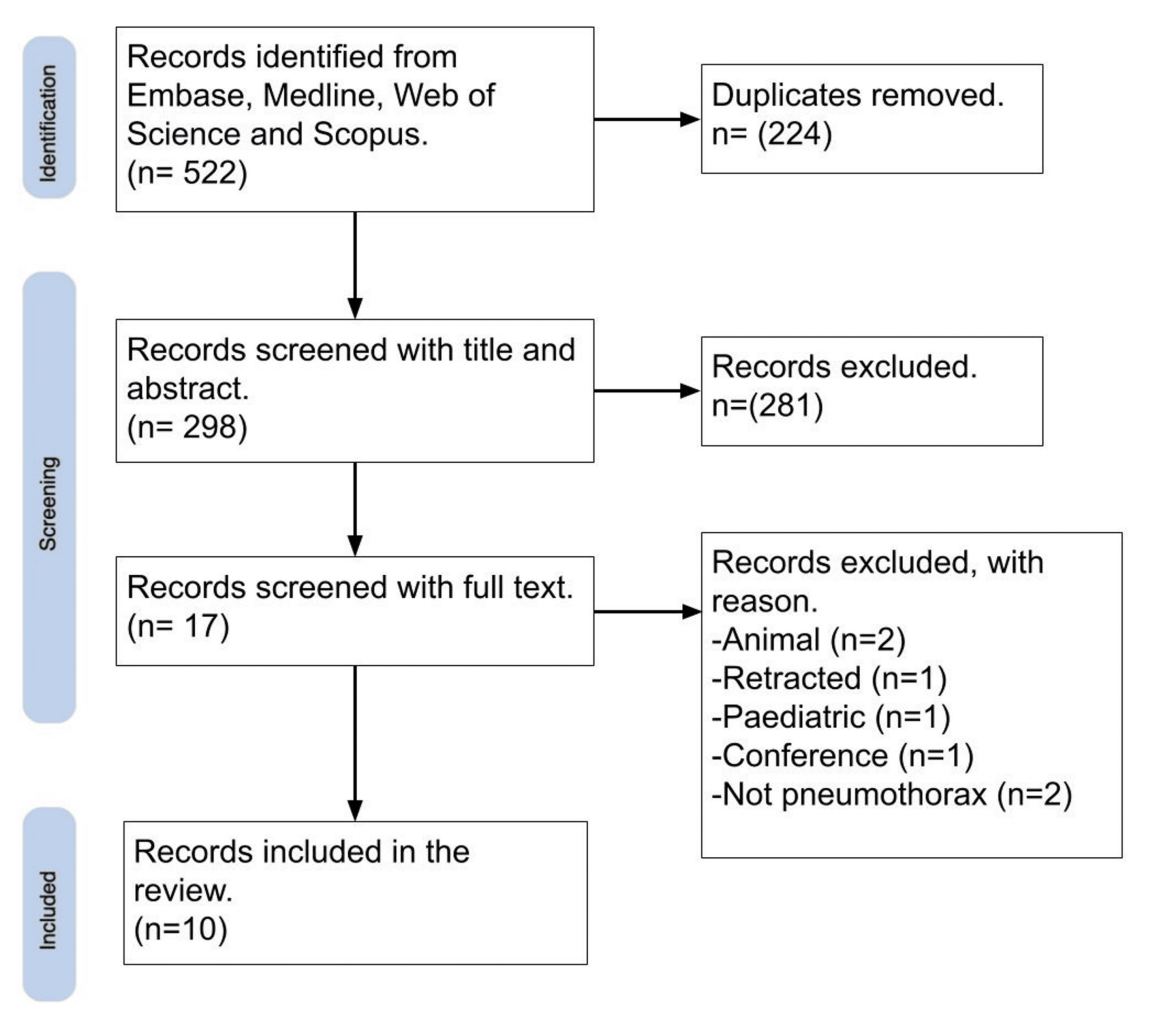
Flowchart of the study selection process

After completion of screening, 10 studies were identified which met the search criteria (Figure 1). The studies span from 2016 to 2024, with nine published after 2020 (Table 1). Eight used a retrospective design, and two studies published in 2024 were prospective. Physician analysis of ultrasound was used as a reference standard in nine of the 10 studies, with only one using CT. 

**Table 1:** Study and testing dataset characteristics NR: Not reported * The two marked studies used the same dataset. Sonosite: FUJIFILM Sonosite, Inc., Bothell, Washington, USA; Sonoscape: SonoScape Medical Corp, Shenzhen, China; Mindray: Mindray Medical International Limited, Shenzhen, China; Philips: Royal Philips, Amsterdam, Netherlands; Konica Minolta: Konica Minolta, Inc., Tokyo, Japan; Vinno: VINNO Technology (Suzhou) Co, Ltd., Suzhou, China

Study characteristics				Testing dataset characteristics (source dataset)					
First author	Year	Retrospective/ prospective	Reference standard	Patient number	Original format + data number (n)	Dataset split (testing/training+validation)	Setting	Ultrasound machine(s)	Ultrasound probe(s)
Summers [16]	2016	Retrospective	Clinician consensus	NR	B-mode (n=107) + M-mode(n=26)	100% testing (separate training dataset)	One hospital (emergency department)	Sonosite M-turbo + Sonosite S-FAST	Linear + phased array
Jascur * [17]	2021	Retrospective	Clinician labelling	48	B-mode (n=48)	25% testing / 75% training + validation	One hospital (post-thoracic surgery patients)	SonoScape S2 Portable	Linear
VanBerlo [18]	2022	Retrospective	Clinician labelling with review	738	B-mode (n=3075)	15% testing / 85% training + validation.	Two hospitals (emergency department + intensive care unit +internal medicine wards)	Mix of machines and manufacturers (Sonosite + Mindray + Philips)	Linear + phased array + curvilinear
Montgomery [19]	2023	Retrospective	Clinician consensus	45	B-mode (n=45)	100% testing (separate training dataset)	NR	Philips Lumify	Curvilinear
Zhang [20]	2023	Retrospective	Clinician labelling	204	M-mode (n=2000)	22.5% testing / 77.5% training + validation	One hospital	Konica Minolta Sonoimage HS1	NR
Kolarik * [21]	2023	Retrospective	Clinician labelling	48	B-mode (n=48)	30% testing / 70% training + validation	One hospital (post-thoracic surgery patients)	SonoScape S2 Portable	Linear
Kim [22]	2023	Retrospective	Clinician consensus	77	B-mode (n=99)	100% testing (separate training dataset)	NR	NR	NR
Yang [23]	2024	Prospective	Computed tomography	75	B-mode (n=NR)	100% testing (separate training dataset)	One hospital (critical care patients with dyspnea)	Vinno 6	Linear + Curvilinear
Fiedler [24]	2024	Prospective	Clinician consensus	62	B-mode (n=241)	100% testing (separate training dataset)	One hospital (thoracic surgical ward + intensive care unit)	Mindray M9	Phased array
Wu [25]	2024	Retrospective	Clinician labelling with review	238	B-mode (n=641)	67% testing / 33% fine-tuning (separate original training data set)	Three hospitals (emergency department + intensive care unit)	Mix of machines and manufactures (Sonosite + Mindray + Philips)	Curvilinear + phased array

All three conventional probe types were used to test algorithms, with linear used five times, curvilinear four, and phased array four. Four of the studies tested their algorithms on more than one probe type [16,18,23,25]. The two prospective studies utilised established ultrasonography protocols. Yang et al. utilised the BLUE (Bedside Lung Ultrasound in Emergency) protocol and Fiedler et al. utilised the E-FAST (Extended Focused Assessment with Sonography for Trauma) protocol [23,24]. The other eight studies did not report specific scanning protocols.

Three studies shared the same diagnostic model [18,24,25]. This was originally developed by VanBerlo et al., and then subsequently tested on different data sets by Wu et al. and Fiedler et al. Wu et al. additionally fine-tuned the model on one-third of their new data set. Four studies performed the final test of their software on the same data set with which it had been trained [17,18,20,21]. Five studies used separate datasets for training and final testing [16,19,22-24]. The studies by Jascur et al. and Kolarik et al. shared the same dataset for both training and testing but with different models [17,21].

Eight studies used convolutional neural networks as the method of classification, with one study using a rules-based algorithm and one study not specifying the type of software (Table 2). The majority of studies performed final classification on M-mode images, with five studies transforming B-mode to M-mode as part of data processing [17,18,22,24,25]. Montgomery et al. performed classification on the standard deviation of pixels, in a technique similar to M-mode [19]. Kolarik et al. performed classification directly on video using a 3D convolutional neural network [21]. Five studies detailed data augmentation methods [17,18,20,22,25], to generate more data for either testing or training.

**Table 2:** Software characteristics CNN: Convolutional neural network; AI: Artificial intelligence; DL: Deep learning; NR: Not reported; ROI: Region of interest *The three marked studies used the same classification model.

First author	Classification software	Additional AI used in the pipeline	Classifier final format	Training dataset
Summers [16]	Diagnostic algorithm (iFAST)	None	M-mode + B-mode	160 videos
Jascur [17]	CNN (ResNet 18)	U-Net CNN for tissue segmentation	M-mode	Same as testing
VanBerlo * [18]	CNN (EfficientNetB0)	Automask DL tool for removal of extraneous information + single-shot detector for ROI detection	M-mode	Same as testing
Montgomery [19]	CNN (2D UNet)	3D dynamic UNet for tissue segmentation	B-mode	30 videos from 30 patients
Zhang [20]	CNN (EfficientNetB0)	CNN for denoising	M-mode	Same as testing
Kolarik [21]	CNN (Resnet 3D)	None	B-mode	Same as testing
Kim [22]	CNN (EfficientNet-lite0)	EfficientNet-lite0 for combined quality assurance classification and ROI detection	M-mode	170 patients from the emergency department
Yang [23]	NR	NR	NR	NR
Fiedler * [24]	CNN (EfficientNetB0)	Automask DL tool for removal of extraneous information + single-shot detector for ROI detection	M-mode	738 Patients from 2 hospitals
Wu * [25]	CNN (EfficientNetB0)	Automask DL tool for removal of extraneous information + single-shot detector for ROI detection	M-mode	738 Patients from 2 hospitals

The studies described a variety of selection and inclusion criteria. Three studies reported patient-level exclusion criteria. Summers et al. excluded one scan for patient age [16]. Yang et al. excluded patients with serious chest trauma, obesity, thoracic malformation, or prior thoracocentesis [23]. Fiedler et al. only excluded patients if their condition prevented ultrasound scanning [24].

Six studies reported exclusion criteria for images or videos [16,18,19,22,24,25]. Kim et al. and VanBerlo et al. reported excess probe movement as an exclusion criterion, while Montgomery et al. excluded the first and last frames of each video to avoid the effects of excess probe movement [18,19,22]. VanBerlo et al. additionally excluded videos with B-lines, consolidations, pleural effusions, and annotations. Kim et al. and Summers et al. both excluded data that was diagnostically indeterminate, while Fiedler et al. created an indeterminate category, which was not included in their primary outcome [16,22,24]. Wu et al. excluded clips acquired with a linear probe [25]. Kim et al. automated some elements of exclusion, building quality assessment into their overall pipeline [22].

The majority of studies selected lung sliding as the primary outcome, with five using its absence and three its presence (Table 3). Yang et al. used pneumothorax, as identified on CT, as the primary outcome [23]. Six studies also considered other ultrasonographic signs of pneumothorax. Zhang et al. used a combined average of three metrics as the primary outcome: the presence of lung sliding, the absence of lung sliding, and the presence of the lung point [20]. Yang et al. utilised the lung point as a positive sign for pneumothorax diagnosis [23]. Kim et al. performed M-Mode classification three times in each region of interest; if there were adjacent positive and negative outcomes consistent with a lung point, this was classified as negative for lung sliding [22]. VanBerlo et al., Fiedler et al., and Wu et al. assigned lung pulse clips to the negative class (presence of sliding) and excluded lung point clips [18,24,25].

**Table 3:** Diagnostic accuracy measures NR: Not reported; AUC: Area under the curve

First author	Primary outcome	Version	Sensitivity	Specificity	Accuracy	AUC
Summers [16]	Presence of lung sliding	Single version tested	0.79	0.87	NR	NR
Jascur [17]	Absence of lung sliding	64 frame version, with centroid point selection	0.82	0.92	0.89	NR
VanBerlo [18]	Absence of lung sliding	Single version tested	0.94	0.87	0.89	0.97
Montgomery [19]	Presence of lung sliding	Single version tested	0.86	0.75	NR	NR
Zhang [20]	Average of lung sliding presence + lung sliding absence + lung point presence	Single version tested	0.98	0.98	NR	0.98
Kolarik [21]	Absence of lung sliding	Resnet3D-18 on 30x30 dataset	0.94	0.79	0.92	NR
Kim [22]	Presence of lung sliding	The whole pipeline, including quality assurance	0.88	0.80	0.84	0.89
Yang [23]	Presence of pneumothorax	Single version tested	0.79	0.85	NR	NR
Fiedler [24]	Absence of lung sliding	Excluding indeterminate clips	0.92	0.80	0.82	0.89
Wu [25]	Absence of lung sliding	Fine-tuned on one-third of the external dataset	0.92	0.82	0.83	0.92

Risk of Bias

QUADAS-2 assessment of the risk of bias showed that all included studies had a high or unclear risk of bias for patient selection, reflecting the retrospective nature and lack of randomised patient sampling of the included studies (Table 4). Four studies were deemed to be high risk for the index test; this corresponds to studies which trained and tested on the same dataset. All studies were deemed low risk in terms of flow and timing, primarily due to the reference standard being the human interpretation of the same images. Overall risk of bias for applicability was low, reflecting the review question being broad.

**Table 4:** QUADAS-2 assessment of risk of bias H: High; L: Low; U: Unclear; QUADAS-2: the revised version of the Quality Assessment of Diagnostic Accuracy Studies

	Risk of bias				Applicability		
First author	Patient selection	Index test	Reference standard	Flow and timing	Patient selection	Index test	Reference standard
Summers [16]	H	L	L	L	L	L	L
Jascur [17]	U	H	H	L	L	L	L
VanBerlo [18]	H	H	L	L	L	L	L
Montgomery [19]	H	L	U	L	L	L	L
Zhang [20]	U	H	H	L	L	L	L
Kolarik [21]	U	H	H	L	L	L	L
Kim [22]	H	L	L	L	L	L	L
Yang [23]	H	L	U	L	L	U	L
Fiedler [24]	H	L	L	L	L	L	L
Wu [25]	H	L	L	L	L	L	L

Discussion

Automated diagnosis of pneumothorax using ultrasound is a fast-moving area, stimulated by the rapid evolution of artificial intelligence technology. While the current evidence is limited, our findings suggest that automated analysis of lung ultrasound can be useful to diagnose and exclude pneumothorax. 

The variability in study designs, datasets, and reporting methods makes comparisons of efficacy challenging. The current evidence is mostly retrospective. The two prospective studies from 2024 represent a positive trend toward more robust validation [23,24]; of note, the model demonstrated by Fiedler et al. operated in real-time and by the bedside. Overall risk of bias for patient selection was high. Testing on larger, randomised patient groups would improve validity and allow examination of the impact of patient-specific factors such as age, sex, and pulmonary comorbidities.

Most included studies focused on detecting the absence of lung sliding as a proxy for pneumothorax. However, this approach has limitations. Firstly, the absence of lung sliding is not pathognomonic for pneumothorax and can occur in other conditions such as acute respiratory distress syndrome and phrenic nerve palsy. Additionally, the interpretation of lung sliding can be subject to interobserver variability, and some cases can be indeterminate. Indeterminate cases were identified in several of the included studies [16,22,24]. Automated ultrasonography therefore requires interpretation in combination with history and examination findings to reliably diagnose pneumothorax, and a role is likely to remain for chest X-rays in certain circumstances. 

This review used the QUADAS-2 assessment tool, which was not designed for studies related to AI. Although the tool was adapted to the review, there are limitations to its use here. Assessment tools specifically designed for AI diagnostics would improve the validity and consistency of bias assessments, and an AI-specific version of QUADAS is currently under development [26].

The breadth of probes and the settings used in lung ultrasonography are limitations for the interoperability of potential automated analysis software. Larger and more diverse training datasets would help to mitigate this. Variability in scanning protocols is not addressed by many of these papers, which focus on lung sliding at a single location. Developing systems that can generalize across diverse hardware and acquisition protocols will aid more widespread clinical adoption. Most studies performed final analysis on M-mode images, which allows the use of simpler models with less computational load, suitable for mobile devices. Progress in model architecture and improvements in computing performance will allow more studies into the direct analysis of video, as demonstrated by Kolarik et al. [21].

Studies varied in their handling of poor-quality images, which biases their results and makes comparisons of accuracy difficult. Ultrasonography differs from other forms of automated imaging analysis in that image acquisition is an intrinsic part of the diagnostic process. Some low-quality scanning is inevitable, particularly in high-intensity environments. The development of complementary software that supports scanning techniques and improves scan quality will be useful for the technology to be accessible to non-experts. Kim et al. demonstrated a quality assessment algorithm as an intrinsic component of their model [22]; this would allow the rejection of inadequate scans at the bedside and therefore improve diagnostic accuracy.

The clinical applications for automated pneumothorax detection vary widely, from trauma assessment to post-procedural monitoring. This diversity necessitates careful consideration of the required performance metrics for each use case. For instance, in resuscitation, high sensitivity may be prioritized to avoid missing a tension pneumothorax. Conversely, for routine post-procedural screening, a better balance of sensitivity and specificity might be desired to minimize false positives. Future research should clearly define the intended clinical application and tailor performance goals accordingly.

The majority of studies employed convolutional neural networks, reflecting the current state of the art in medical image analysis. However, the field of AI is rapidly evolving, with newer architectures like vision transformers showing promise in other medical imaging domains. Future research should explore the applicability of these emerging technologies to lung ultrasound interpretation.

## Conclusions

Automated analysis of lung ultrasound for pneumothorax diagnosis is a promising technology. However, conclusive evidence for the efficacy of this technology is currently lacking due to limited prospective trials and methodological heterogeneity. Future research should focus on larger, more diverse datasets, standardized evaluation metrics, and robust external validation. Consideration could be given to trialling new AI techniques.

## Appendices

Search strategy

Two search strategies were used in each database and combined. One search was conducted for the combination of ultrasound and pneumothorax and automated diagnosis. The other search was for the combination of automated diagnosis and lung sliding or the lung point. Relevant mesh terms and keywords for each database were also included.

The below terms were used to search for the concepts for ultrasound and automated diagnosis. The examples are in the MEDLINE format.

Ultrasound

ultrasound or sonographic or sonography or lung ultrasound or LUS or ultrasonography

Automated Diagnosis

((artificial adj3 intelligen*) or (artificially adj3 intelligen*) or ai or ani or cad or machine learning or deep learning or computer vision or machine vision or image recognition or ((neural or convolutional or deconvolutional or recurrent or feed forward or deep) adj3 (network or networks or net or nets)) or ((model or learning or training or pretraining) adj3 (supervised or semi-supervised or reinforcement or unsupervised)) or ((diagnosis or diagnostic or diagnostics or diagnostic assistant or diagnostic assistance or identification or interpretation or detection or recognition or assessment or exclusion or analysis or classification or decision support or decision making) adj3 (automated or automation or autonomous or automatic or software or model or computerised or computer or computational or digital or algorithm or algorithmic or programme))
